# Cross-over study of influence of oral vitamin C supplementation on inflammatory status in maintenance hemodialysis patients

**DOI:** 10.1186/1471-2369-14-252

**Published:** 2013-11-14

**Authors:** KunYing Zhang, YinHui Li, XuYang Cheng, Li Liu, WenYing Bai, WeiYa Guo, LeiYun Wu, Li Zuo

**Affiliations:** 1 Department of Medicine, Renal Division, Peking University First Hospital, Beijing, P. R. China; 2 Peking University Institute of Nephrology, Beijing, P. R. China; 3 Key Laboratory of Renal Disease, Ministry of Health of China, Beijing, P. R. China; 4 Department of Nephrology, Peking University People’s Hospital, Beijing, P. R. China; 5 Jilin University Fourth Hospital, Changchun, Jilin, P. R. China; 6 Beijing Daxing district People’s Hospital, Beijing, P. R. China; 7 Guangwai Hospital, Beijing, P. R. China; 8 Beijing No. 2 Hospital, Beijing, P. R. China

**Keywords:** Hemodialysis, Inflammation, Vitamin C

## Abstract

**Background:**

Both vitamin C deficiency and inflammation are prevalent in maintenance hemodialysis (MHD) patients. In this study, we aimed to elucidate the effect of oral vitamin C supplementation on inflammatory status in MHD patients with low vitamin C level and high hypersensitive C-reactive protein (hs-CRP) level.

**Methods:**

A total of 128 patients were recruited in our present study. Patients were divided into two groups. In group 1 (n = 67), patients were orally administered with 200 mg/day vitamin C in the first 3 months, and then the vitamin C supplementation was withdrawn in the next 3 months. In group 2 (n = 61), patients were not given vitamin C in the first 3 months, and then they were orally administered with 200 mg/day in the next 3 months. Levels of hs-CRP, prealbumin, albumin and hemoglobin as well as the EPO resistance index (ERI) were determined at the baseline and every 3 months throughout the study. Plasma vitamin C level was determined by high-performance liquid chromatography with UV detection.

**Results:**

Among the 128 patients, 28 of them dropped out of the study before completion. Consequently, a total of 100 patients (group 1: n = 48; group 2: n = 52) were included in the final analysis. At the baseline, the plasma vitamin C level of all patients was less than 4 μg/mL. However, this proportion was decreased to 20% after the vitamin C supplementation for 3 months. Compared with patients without the vitamin C supplementation, a decreased level of hs-CRP and an increased level of prealbumin were induced by the vitamin C supplementation for 3 months in both groups. However, levels of these biomarkers returned to their original state after the supplementation was withdrawn. Same beneficial effects on plasma albumin, hemoglobin and ERI response to vitamin C supplementation were observed in the two groups without statistical significance.

**Conclusions:**

The inflammatory status in MHD patients with plasma vitamin C deficiency and high levels of inflammatory markers could be partially improved by long-term oral administration of small doses of vitamin C.

**Trial registration:**

The clinical trial number: NCT01356433.

## Background

The life expectancy of maintenance hemodialysis (MHD) patients is significantly lower than that of healthy subjects [1]. Atherosclerotic cardiovascular disease is the leading cause of death in this population. In addition to traditional cardiovascular risk factors, there are some non-traditional risk factors, including micro-inflammation [2,3]. Therefore, measures should be taken to correct the conditions with a possibly negative effect on arthrosclerosis.

Inflammation is prevalent in MHD patients. As an inflammatory marker, C-reactive protein (CRP) is an important sensitive acute phase reactant [4]. Previous investigations demonstrated that high level of serum CRP is a strong predictor of cardiovascular mortality both in healthy subjects [5] and MHD patients [6].

Vitamin C is one of the most important water soluble antioxidants. It is well established that the plasma vitamin C level is generally lower in MHD patients [7,8] than that in general population, which is attributed to inadequate dietary intake, oxidative stress [9] and loss during dialysis session [7,10].

The low level of plasma vitamin C as well as inflammatory status has been recently reported to be closely related to the increased risk of cardiovascular morbidity and mortality in either MHD or peritoneal dialysis (PD) patients [6,11]. Our previous cross-sectional analysis showed that low level of plasma vitamin C is negatively associated with the CRP level [12].

The hypothesis that the inflammatory status may be improved by vitamin C supplementation has been studied in a limited number of investigations based on a limited number of patients, resulting in conflicting results. One study was conducted on 33 MHD patients for 2 months [13], another one was conducted on 20 MHD patients for 2 months [14], and both studies did not get positive conclusions. However, an investigation documented that the 8-hydroxy-2′-deoxyguanosine (8-OHdG) level of cellular DNA is reduced after the vitamin C supplementation for 8 weeks in chronic hemodialysis patients [15]. These previous conflicting results might be partly due to either limited sample size or short period of observation.

In the present study, we designed a randomized controlled cross-over study with relatively large sample size and aimed to investigate the effect of vitamin C supplementation on inflammatory status in MHD patients.

## Methods

### Study patients

The effect of oral vitamin C supplementation on inflammatory status in MHD patients with low vitamin C level and high hypersensitive CRP (hs-CRP) level was investigated using a randomized controlled cross-over study. Patients who met all of the following inclusion criteria were included: (1) more than 18 years old; (2) under stable condition, receiving conventional hemodialysis for 4–4.5 hours thrice weekly and MHD for at least 3 months; Kt/V > 1.2; (3) plasma vitamin C level < 4 μg/mL and hs-CRP level > 3 mg/L; (4) not receiving any form of vitamin C supplementation within 3 months prior to the investigation. Patients with any one or more exclusion criteria were excluded from the investigation: (1) either hepatitis B surface antigen positive, hepatitis C antibody positive or HIV carrier; (2) acute infection within 1 month prior to the investigation; (3) neoplasm, hemopathy or active autoimmune disease; (4) use of steroids and/or immunosuppressive agents within 3 months prior to the investigation; (5) pregnancy or breast feeding.

In the present study, 128 MHD patients were recruited from five dialysis facilities in North China. The mean age and the mean dialysis vintage of the patients were 64.1 ± 12.1 years and 50.6 ± 32.5 [median 48, inter-quartile range (IQR) 21, 72] months, respectively. Patients were divided into two groups as follows. In group 1 (n = 67), patients were orally administered with 200 mg/day vitamin C in the first 3 months, and then the vitamin C supplementation was withdrawn in the next 3 months. In group 2 (n = 61), patients were not given vitamin C in the first 3 months, and then they were orally administered with 200 mg/day vitamin C in the next 3 months. No patient was provided with omega-3 and/or vitamin E. Levels of plasma vitamin C, hs-CRP, prealbumin, albumin and biochemical parameters of interest were determined at the baseline and every 3 months throughout the study.

This study was approved by the Ethics Committee of Clinical Research, Peking University First Hospital (clinical trial number: NCT01356433). Written informed consent was obtained from all participants.

### Sample collection and laboratory measurements

Fasting blood samples were collected from MHD patients through the arteriovenous fistula just before dialysis session. Collected blood samples were transported to the laboratory using heparin-containing tubes in an ice bath. Plasma was separated by centrifugation (2,000 g, 10 min) at 4°C within 30 min. An aliquot of 200 μL plasma was immediately mixed with 200 μL of 10% metaphosphoric acid (MPA) due to the instability of vitamin C in plasma, and the mixture was then stored at −80°C until further analysis within 2 weeks.

Vitamin C level was determined by high-performance liquid chromatography (HPLC) (Agilent 1100 series, Agilent Technologies, USA) on a Diamonsil C18 column (150 mm × 4.6 mm, 3 μm) with UV detection according to the previously described method [12,16]. Intra-assay and inter-assay coefficients of variation were 2.7% and 2.5%, respectively. The reference vitamin C level in normal population ranges from 4 to 14 μg/mL [17,18].

EPO resistance index (ERI) was defined as the ratio of the dosage of recombinant human erythropoietin (rHuEpo) (IU/kg/week) and the concentration of hemoglobin (g/dl) on the day of plasma sampling. The hs-CRP level was determined using the immunonephelometric assay (First Chemical Pharmaceutical Co., Japan) with a detection limit of 0.1 mg/L. Levels of prealbumin, albumin, ferritin, calcium, serum bicarbonate, uric acid and hemoglobin were determined by standard techniques in the Clinical Laboratory of Peking University First Hospital, Beijing, China.

Patient baseline demographics, including age, gender, primary cause of end stage renal disease (ESRD), dialysis vintage and drug use, were also collected and recorded.

### Statistical analysis

Normally distributed variables, such as albumin, prealbumin and hemoglobin, were expressed as mean ± SD. Non-normally distributed variables, such as vitamin C and hs-CRP, were presented as median and IQR. Levels of albumin, prealbumin and hemoglobin between groups were compared using Student’s *t*-test or one-way analysis of variance (one-way-ANOVA). Categorical variables between groups were compared using *χ*^2^ test. Levels of vitamin C and hs-CRP between groups were compared using Mann–Whitney test or Kruskal Wallis Test. Statistical analysis was performed using SPSS version 11.5 (SPSS, Inc., Chicago, IL, USA). A P value of less than 0.05 was considered as statistically significant.

## Results

### Demographics

Among the 128 patients, 28 of them dropped out of the study before completion as follows: four heart failure, three acute infection, two repeated hemafecia, two pancytopenia of unknown reason, one renal transplant, one bladder carcinoma, five non-compliance, one wound and surgery, two transferred to other facilities and seven death. Consequently, a total of 100 patients (group 1: n = 48; group 2: n = 52) were included in the final analysis (47 males, 53 females), with a mean age of 64.4 ± 11.7 years and a median dialysis vintage of 48 (IQR 21, 72) months (Table 1).

**Table 1:** Baseline characteristics of the study population (n = 100)

**Item**	**Group 1**	**Group 2**	**p value**
	**(n = 48)**	**(n = 52)**	
Age (year)	64.3 ± 11.7	64.4 ± 11.8	0.962
gender			0.029
Male	17(35.4%)	30(57.7%)	
female	31(65.6%)	22(42.3%)	
BMI (kg/m^2^)	21.9 ± 3.3	22.7 ± 3.3	0.248
KT/V	1.6 ± 0.2	1.5 ± 0.3	0.551
Dialysis vintage (m)	56.9 ± 32.2	44.8 ± 32.0	0.062
Diabetes	9/39(23.1%)	11/41(21.2%)	0.807
ACEI/ARB (case)	6/48(12.5%)	6/52(11.5%)	1.000
Statins (case)	2/48(4.2%)	3/52(5.8%)	1.000
BUN (mmol/l)	25.2 ± 5.6	24.9 ± 7.3	0.884
Scr (umol/l)	783.9 ± 179.7	920.1 ± 256.9	0.058
prealbumin (mg/l)	295.6 ± 86.6	315.3 ± 85.8	0.256
albumin (g/l)	38.2 ± 3.7	40.0 ± 4.2	0.023
hemoglobin (g/l)	107.2 ± 16.6	111.4 ± 17.3	0.217
ERI	10.7 ± 8.6	9.3 ± 6.1	0.323
Ferritin (ug/l)	417.8 ± 266.5	461.9 ± 287.1	0.429
Vitamin C (μg/mL)	1.5 ± 0.8	2.0 ± 0.9	0.003
	Median(IQR)	Median(IQR)	
hsCRP(mg/l)	9.6 (6.0-13.8)	6.2(4.2-11.0)	0.005

Note: age, BMI, dialysis vintage, BUN, Scr, prealbumin, albumin, hemoglobin, ERI and vitamin C were presented as mean ± SD; hsCRP was presented as median and inter-quartile range (IQR).

*Abbreviations*: *BMI* body mass index, *BUN* blood urea nitrogen, *Scr* serum creatinine, *ERI* EPO resistance index, *hs-CRP* high hypersensitive C-reactive protein, *IQR* inter quartile range.

Among the 100 patients, the primary causes of ESRD were chronic glomerulonephritis (n = 20), interstitial nephropathy (n = 20), diabetic nephropathy (n = 20), hypertensive nephrosclerosis (n = 18), polycystic disease (n = 11), chronic pyelonephritis (n = 3) and others (n = 8). Table 1 lists the detailed demographics.

Patients in group 2 exhibited lower hs-CRP level, higher vitamin C and albumin levels compared with group 1. Moreover, there were more males in group 2. No statistical significance was found in terms of age, gender, body mass index, dialysis vintage, Kt/V, prealbumin, hemoglobin and ERI between two groups.

### Change of vitamin C level during the study

At the baseline, the plasma vitamin C level of all patients was less than 4 μg/mL. However, this proportion was decreased to 20% after the vitamin C supplementation for 3 months.

**Figure 1:**
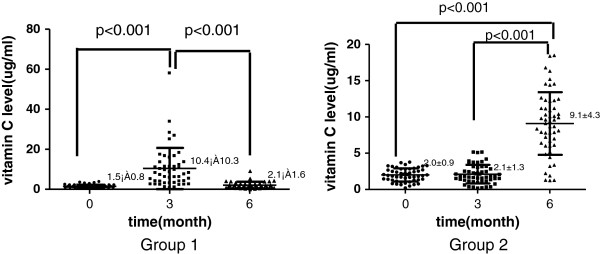
**Influence of vitamin C supplementation on plasma vitamin C level.** group1: patients were given oral vitamin C 200 mg per day during the first 3 months and withdraw vitamin C thereafter. group2: patients were given vitamin C during the second 3 months; vitamin C was presented as mean ± SD; levels of vitamin C were compared among groups using one-way analysis of variance (1-way-ANOVA).

For group 1, the vitamin C level was significantly increased at the end of the first 3 months (p < 0.001) compared with that at the baseline. After the vitamin C supplementation was withdrawn, the vitamin C level was significantly decreased at the end of the second 3 months compared with that at the end of the first 3 months (p < 0.001). Moreover, no significant difference in the vitamin C level was found between the end of the second 3 months and the baseline (p = 0.606) (Figure 1).

For group 2, the vitamin C level remained unchanged at the end of the first 3 months (p = 0.837) compared with that at the baseline. However, it was significantly increased at the end of the second 3 months compared with that at the baseline (p < 0.001) and the end of the first 3 months (p < 0.001) (Figure 1).

### Change of hs-CRP level during the study

**Figure 2:**
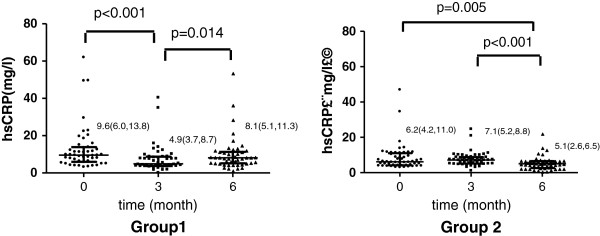
**Effect of vitamin C supplementation on plasma hs-CRP level.** Group 1: patients were orally administered with 200 mg/day vitamin C during the first 3 months, and the vitamin C administration was withdrawn thereafter. Group 2: patients were administered with vitamin C during the second 3 months. Hs-CRP level was presented as Median (IQR); levels of hs-CRP were compared among groups using Kruskal Wallis Test.

For group 1, the hs-CRP level was significantly decreased at the end of the first 3 months (p < 0.001) compared with that at the baseline. After the vitamin C supplementation was withdrawn, the hs-CRP level was significantly increased at the end of the second 3 months compared with that at the end of the first 3 months (p = 0.014). Moreover, no significant difference in the hs-CRP level was found between the end of the second 3 months and the baseline (p = 0.106) (Figure 2).

For group 2, the hs-CRP level remained unchanged at the end of the first 3 months (p = 0.663) compared with that at the baseline. However, it was significantly decreased at the end of the second 3 months compared with that at the baseline (p = 0.005) and the end of the first 3 months (p < 0.001) (Figure 2).

### Change of prealbumin and albumin levels during the study

For group 1, levels of prealbumin and albumin exhibited a slight increase (p > 0.05) at the end of the first 3 months compared with those at the baseline. After the vitamin C supplementation was withdrawn, levels of prealbumin and albumin (p > 0.05) were decreased at the end of the second 3 months (Table 2).

**Table 2:** Effect of vitamin C supplementation on parameters during 6 months (n = 100)

**Item**	**Group 1 (n = 48)**			**Group 2 (n = 52)**		
	**Baseline**	**Month 3**	**Month 6**	**Baseline**	**Month 3**	**Month 6**
prealbumin (mg/l)	295.6 ± 86.6	296.7 ± 60.1	272.1 ± 69.3	315.3 ± 85.8	302.9 ± 60.3	336.9 ± 69.5^a^
albumin (g/l)	38.2 ± 3.7	38.3 ± 3.1	37.6 ± 2.6	40.0 ± 4.2	39.6 ± 2.8	40.4 ± 2.4
hemoglobin (g/l)	107.2 ± 16.6	109.9 ± 14.1	109.3 ± 14.2	111.4 ± 17.3	110.9 ± 20.4	111.9 ± 25.4
ERI	10.7 ± 8.6	8.3 ± 6.6	9.1 ± 7.4	9.3 ± 6.1	8.6 ± 6.7	7.5 ± 6.4
Ferritin (ug/l)	417.8 ± 266.5	355.0 ± 256.4	444.2 ± 333.5	461.9 ± 287.1	500.0 ± 314.2	445.9 ± 352.7
Vitamin C (μg/mL)	1.5 ± 0.8	10.4 ± 10.3^b^	2.1 ± 1.6^c^	2.0 ± 0.9	2.1 ± 1.3	9.1 ± 4.3^bc^
		Median(IQR)			Median(IQR)	
rHuEpo (x10^3^U/week)	6.0(3.0-9.0)	4.5(2.8-6.0)	5.2(2.6-8.8)	6.0(4.0-8.8)	6.0(3.0-8.8)	4.2(2.7-6.0)
hsCRP (mg/l)	9.6 (6.0-13.8)	4.9(3.7-8.7)^b^	8.1(5.1-11.3)^d^	6.2(4.2-11.0)	7.1(5.2-8.8)	5.1(2.6-6.5)^bc^

Note: ^a^p < 0.05:compared with group baseline; ^b^p < 0.01:compared with group baseline; ^c^p < 0.01:compared with group Month 3; ^d^p < 0.05:compared with group Month 3; prealbumin, albumin, hemoglobin, ERI, ferritin and vitamin C levels were presented as mean ± SD; EPO dosage and hs-CRP level were presented as median and inter-quartile range (IQR).

*Abbreviations*: *ERI* EPO resistance index, *rHuEpo*, recombinant human erythropoietin, *hs-CRP* high hypersensitive C-reactive protein, *IQR* inter quartile range.

For group 2, levels of prealbumin and albumin remained unchanged at the end of the first 3 months (p > 0.05) compared with those at the baseline. However, a significant increase in prealbumin (p = 0.018) and an increase trend in albumin (p > 0.05) were observed at the end of the second 3 months compared with those at the end of the first 3 months (Table 2).

### Change of ERI during the study

For group 1, a decrease trend in ERI, ferritin and EPO dosage (all p > 0.05) and an increase trend in hemoglobin (p > 0.05) were observed at the end of the first 3 months compared with those at the baseline. At the end of the second 3 months, ERI, ferritin and EPO dosage (all p > 0.05) were increased without statistical significance compared with those at the end of the first 3 months, whereas the hemoglobin level remained unchanged at the end of the second 3 months (Table 2).

For group 2, a decrease trend in ERI and hemoglobin and an increase trend in ferritin were observed at the end of the first 3 months compared with those at the baseline (all p > 0.05), whereas the EPO dosage remained unchanged. At the end of the second 3 months, a decrease trend in ERI, ferritin and EPO dosage and an increase trend in hemoglobin were observed (all p > 0.05) compared with those at the end of the first 3 months (Table 2).

## Discussion

In the present study, we showed that the plasma hs-CRP level in MHD patients could be reduced by oral vitamin C supplementation. The proportion of patients with a plasma vitamin C level of less than 4 μg/mL was decreased to 20% after the vitamin C supplementation for 3 months. We also found an increase trend in plasma prealbumin level after the vitamin C supplementation. In addition, a better plasma albumin, hemoglobin, EPO dosage and ERI response to vitamin C supplementation was observed without statistical significance.

Previous study demonstrated that MHD patients have remarkably low plasma vitamin C levels, frequently < 10 μM, even < 2 μM [8,19]. In our previous study, a plasma vitamin C level of < 4 μg/mL (22.8 μmol/L) is presented in 64.4% dialysis patients [12]. In our current study, 20% patients still exhibited a persistent low plasma vitamin C level after the vitamin C supplementation for 3 months, suggesting that an individualized dosage of vitamin C supplementation should be considered.

Low-level, persistent inflammation is prevalent in MHD patients, although there is no convincing evidence of systemic or restricted infection in clinical practice. Vitamin C deficiency is caused by inadequate dietary intake, loss during dialysis procedure, impaired metabolism and reduced tubular reabsorption [7,10,20-22].

Miyata and Wang S. et al. observed that the concentration of *in vitro* plasma ascorbic acid in uremic patients is decreased more rapidly (0.16% per min) than that in normal subjects (0.09% per min) [23,24]. This finding suggested that the uremic plasma consumes more vitamin C than healthy plasma, which may be related to excessive toxin retention and metabolic acidosis [25]. *In vivo*, the volume overload [26] and bio-incompatibility of dialysis materials and non-sterile dialysate may also contribute to the inflammatory status [27].

In our previous cross-sectional study, we found that a negative correlation existed between the plasma vitamin C level and inflammation status in MHD patients [12]. We hypothesized that vitamin C, as an electron donor, had anti-oxidative effects, and its oral supplementation could improve the inflammatory status in MHD patients. Tarng D C et al. [28] reported that the 8-OHdG level of cellular DNA, as an evaluative indicator of oxidative DNA damage in reactive oxygen species-mediated diseases [15], is reduced after the vitamin C supplementation for 8 weeks in chronic hemodialysis patients. However, this beneficial effect in MHD patients has not been reported by other studies. In Fumeron’s study [13], 33 MHD patients were orally administered with 250 mg vitamin C thrice weekly after each dialysis session for 2 months, and no evident improvement is observed in oxidative/anti-oxidative stress and inflammation markers. Kamgar M et al. [14] reported a decrease trend in CRP level after an oral supplementation of 250 mg/day vitamin C for 2 months in 20 MHD patients. In our present study, the hs-CRP level was decreased by oral supplementation of 200 mg/day vitamin C in both groups, and the hs-CRP level was increased again after the vitamin C supplementation was withdrawn in group 1. Unlike other inconclusive results from previous studies, we showed that the vitamin C supplementation doubtlessly had a beneficial effect. Our results were more convincing due to following advantages: (1) relative larger sample size; (2) relative longer period of observation; (3) randomized controlled cross-over design; (4) more importantly, selected patients were with low vitamin C level and high hs-CRP level, and this patient population might respond well to inflammation-induced vitamin C consumption. In this study, several patients took anti-inflammatory drugs, such as ACEI/ARB, statins, but remain unchanged during the study period. Therefore, the anti-inflammatory effects of these drugs on our patients could be sagely ignored.

Recent evidence showed that the plasma vitamin C level is positively associated with levels of hemoglobin [29], albumin [30] and prealbumin [12], and negatively associated with ERI [31-33]. After 6 months of vitamin C supplementation, levels of prealbumin, albumin and hemoglobin are significantly increased in the preliminary study. In the present randomized controlled cross-over study, we also found beneficial responses of these markers upon the vitamin C supplementation, but statistically insignificant, which could be due to the long half-life of serum albumin and hemoglobin, and the short interventional duration. These beneficial effects might be caused by anti-oxidative effect of vitamin C.

Consistent with our data, previous study showed that the vitamin C supplementation improves the responsiveness to EPO in hemodialysis patients with refractory anemia and hyperferritinemia [31]. In our present study, a decrease trend in ERI, ferritin and EPO dosage, and an increase trend in hemoglobin were observed after the oral vitamin C supplementation for 3 months. One possible mechanism for this effect might be the electron offering ability of vitamin C. Vitamin C mobilizes storage iron by reducing ferric iron (Fe^+3^) to ferrous iron (Fe^+2^), including the portion of tissue iron as hemosiderin [34], leading to an increased bioavailability of iron and improved red blood cell production. In the present study, the improvement in hemoglobin was associated with significantly decreased hs-CRP levels during the vitamin C supplementation, but not in controls, which might be due to the anti-oxidative ability of vitamin C.

In this study, the prealbumin concentration was significantly increased after the oral vitamin C administration in group 2 but not in group 1, and ERI was decreased in group 2 even in without-drug phase. Therefore, some other important factors, in addition to the relatively short interventional duration, were probably not included in the present investigation.

In order to minimize the possible accumulation of oxalate in patients, the dosage of vitamin C was selected as 200 mg/day. Dosages as high as 500 to 1,000 mg/day for 3 or more than 3 weeks induce significantly increased plasma oxalate levels [35,36]. Our present study had some limitations as follows. (1) The duration of the intervention was relatively short, although changes in hs-CRP level were observed. Changes of albumin, prealbumin, hemoglobin, EPO dosages and ERI were not significant due to their relatively longer half-life, because duration of 3 months only permitted one red blood cell life-span to reach steady state [37]. (2) A total of 28 (21.9%) patients dropped out during the observation, which might result in the imbalance of parameters between the two groups. (3) This investigation did not contain the placebo in the control group.

## Conclusion

In conclusion, our cross-over study indicated that the inflammatory status in MHD patients with plasma vitamin C deficiency and high levels of inflammatory markers could be partially improved by long-term oral administration of small doses of vitamin C. A multi-center randomized controlled study with relatively larger sample size is required to confirm the role of vitamin C in improving the micro-inflammatory state in MHD patients. Moreover, further study is also necessary to assess the long-term outcome of oral vitamin C supplementation in MHD patients.

## Competing interests

The authors declare that they have no competing interests.

## Authors’ contributions

ZKY participated in the design of the study, sampling procedure, and drafted the manuscript. LYH, CXY, LL, BWY, GWY and WLY participated in the design of the study and sampling procedure. ZL conceived the study, and participated in its design and coordination and performed statistical analysis. All authors read and approved the final manuscript.

## Pre-publication history

The pre-publication history for this paper can be accessed here:

http://www.biomedcentral.com/1471-2369/14/252/prepub

## References

[B1] Foley RN Parfrey PS Sarnak MJ Clinical epidemiology of cardiovascular disease in chronic renal disease Am J Kidney Dis 1998 32 5 Suppl 3 S112 S119 9820470 10.1053/ajkd.1998.v32.pm9820470

[B2] Yeun JY Levine RA Mantadilok V Kaysen GA C-reactive protein predicts all-cause and cardiovascular mortality in hemodialysis patients Am J Kidney Dis 2000 35 3 469 476 10.1016/S0272-6386(00)70200-9 10692273

[B3] Zoccali C Cardiovascular risk in uraemic patients-is it fully explained by classical risk factors? Nephrol Dial Transplant 2000 15 4 454 457 10.1093/ndt/15.4.454 10727537

[B4] Owen WF Lowrie EG C-reactive protein as an outcome predictor for maintenance hemodialysis patients Kidney Int 1998 54 2 627 636 10.1046/j.1523-1755.1998.00032.x 9690231

[B5] Ridker PM Cushman M Stampfer MJ Tracy RP Hennekens CH Inflammation, aspirin, and the risk of cardiovascular disease in apparently healthy men N Engl J Med 1997 336 14 973 979 10.1056/NEJM199704033361401 9077376

[B6] Zimmermann J Herrlinger S Pruy A Metzger T Wanner C Inflammation enhances cardiovascular risk and mortality in hemodialysis patients Kidney Int 1999 55 2 648 658 10.1046/j.1523-1755.1999.00273.x 9987089

[B7] Morena M Cristol JP Bosc JY Tetta C Forret G Leger CL Delcourt C Papoz L Descomps B Canaud B Convective and diffusive losses of vitamin C during haemodiafiltration session: a contributive factor to oxidative stress in haemodialysis patients Nephrol Dial Transplant 2002 17 3 422 427 10.1093/ndt/17.3.422 11865087

[B8] Wang S Eide TC Sogn EM Berg KJ Sund RB Plasma ascorbic acid in patients undergoing chronic haemodialysis Eur J Clin Pharmacol 1999 55 7 527 532 10.1007/s002280050668 10501823

[B9] Washio K Inagaki M Tsuji M Morio Y Akiyama S Gotoh H Gotoh T Gotoh Y Oguchi K Oral vitamin C supplementation in hemodialysis patients and its effect on the plasma level of oxidized ascorbic acid and Cu/Zn superoxide dismutase, an oxidative stress marker Nephron Clin Pract 2008 109 2 c49 c54 10.1159/000137628 18544955

[B10] Sullivan JF Eisenstein AB Ascorbic acid depletion during hemodialysis JAMA 1972 220 13 1697 1699 10.1001/jama.1972.03200130029006 5067604

[B11] Paniagua R Frias Y de Ventura MJ Rodriguez E Hurtado ME Alcantara G Vazquez R Ortiz R Salcedo M Rios ME C-reactive protein and anti-Chlamydia pneumoniae antibodies as risk factors of cardiovascular death in incident patients on peritoneal dialysis Perit Dial Int 2003 23 2 132 137 12713079

[B12] Zhang K Liu L Cheng X Dong J Geng Q Zuo L Low levels of vitamin C in dialysis patients is associated with decreased prealbumin and increased C-reactive protein BMC Nephrol 2011 12 18 10.1186/1471-2369-12-18 21548917 PMC3112084

[B13] Fumeron C Nguyen-Khoa T Saltiel C Kebede M Buisson C Drueke TB Lacour B Massy ZA Effects of oral vitamin C supplementation on oxidative stress and inflammation status in haemodialysis patients Nephrol Dial Transplant 2005 20 9 1874 1879 10.1093/ndt/gfh928 15972322

[B14] Kamgar M Zaldivar F Vaziri ND Pahl MV Antioxidant therapy does not ameliorate oxidative stress and inflammation in patients with end-stage renal disease J Natl Med Assoc 2009 101 4 336 344 19397224 10.1016/s0027-9684(15)30881-6

[B15] Kasai H Analysis of a form of oxidative DNA damage, 8-hydroxy-2′-deoxyguanosine, as a marker of cellular oxidative stress during carcinogenesis Mutat Res 1997 387 3 10.1016/s1383-5742(97)00035-5 9439711

[B16] Zhang K Dong J Cheng X Bai W Guo W Wu L Zuo L Association between vitamin C deficiency and dialysis modalities Nephrology (Carlton) 2012 17 5 452 457 10.1111/j.1440-1797.2012.01595.x 22404236

[B17] Reuler JB Broudy VC Cooney TG Adult scurvy JAMA 1985 253 6 805 807 10.1001/jama.1985.03350300093027 3968818

[B18] Richter A Kuhlmann MK Seibert E Kotanko P Levin NW Handelman GJ Vitamin C deficiency and secondary hyperparathyroidism in chronic haemodialysis patients Nephrol Dial Transplant 2008 23 6 2058 2063 10.1093/ndt/gfn084 18353890

[B19] Jackson P Loughrey CM Lightbody JH McNamee PT Young IS Effect of hemodialysis on total antioxidant capacity and serum antioxidants in patients with chronic renal failure Clin Chem 1995 41 8 Pt 1 1135 1138 7628087

[B20] Alkhunaizi AM Chan L Secondary oxalosis: a cause of delayed recovery of renal function in the setting of acute renal failure J Am Soc Nephrol 1996 7 11 2320 2326 8959621 10.1681/ASN.V7112320

[B21] Durose CL Holdsworth M Watson V Przygrodzka F Knowledge of dietary restrictions and the medical consequences of noncompliance by patients on hemodialysis are not predictive of dietary compliance J Am Diet Assoc 2004 104 1 35 41 10.1016/j.jada.2003.10.016 14702581

[B22] Boeschoten EW Schrijver J Krediet RT Schreurs WH Arisz L Deficiencies of vitamins in CAPD patients: the effect of supplementation Nephrol Dial Transplant 1988 3 2 187 193 3140085

[B23] Wang S Schram IM Sund RB Determination of plasma ascorbic acid by HPLC: method and stability studies Eur J Pharm Sci 1995 3 4 231 239 10.1016/0928-0987(95)00011-2

[B24] Miyata T Wada Y Cai Z Iida Y Horie K Yasuda Y Maeda K Kurokawa K van Ypersele DSC Implication of an increased oxidative stress in the formation of advanced glycation end products in patients with end-stage renal failure Kidney Int 1997 51 4 1170 1181 10.1038/ki.1997.160 9083283

[B25] Bellocq A Suberville S Philippe C Bertrand F Perez J Fouqueray B Cherqui G Baud L Low environmental pH is responsible for the induction of nitric-oxide synthase in macrophages. Evidence for involvement of nuclear factor-kappaB activation J Biol Chem 1998 273 9 5086 5092 10.1074/jbc.273.9.5086 9478960

[B26] Demirci MS Demirci C Ozdogan O Kircelli F Akcicek F Basci A Ok E Ozkahya M Relations between malnutrition-inflammation-atherosclerosis and volume status. The usefulness of bioimpedance analysis in peritoneal dialysis patients Nephrol Dial Transplant 2011 26 5 1708 1716 10.1093/ndt/gfq588 20921295

[B27] Stenvinkel P Alvestrand A Inflammation in end-stage renal disease: sources, consequences, and therapy Semin Dial 2002 15 5 329 337 10.1046/j.1525-139X.2002.00083.x 12358637

[B28] Tarng DC Liu TY Huang TP Protective effect of vitamin C on 8-hydroxy-2′-deoxyguanosine level in peripheral blood lymphocytes of chronic hemodialysis patients Kidney Int 2004 66 2 820 831 10.1111/j.1523-1755.2004.00809.x 15253739

[B29] Finkelstein FO Juergensen P Wang S Santacroce S Levine M Kotanko P Levin NW Handelman GJ Hemoglobin and plasma vitamin C levels in patients on peritoneal dialysis Perit Dial Int 2011 31 1 74 79 10.3747/pdi.2009.00154 20558814 PMC3487381

[B30] Lee EJ Myint CC Tay ME Yusuf N Ong CN Serum ascorbic acid and protein calorie malnutrition in continuous ambulatory peritoneal dialysis patients Adv Perit Dial 2001 17 219 222 11510280

[B31] Attallah N Osman-Malik Y Frinak S Besarab A Effect of intravenous ascorbic acid in hemodialysis patients with EPO-hyporesponsive anemia and hyperferritinemia Am J Kidney Dis 2006 47 4 644 654 10.1053/j.ajkd.2005.12.025 16564942

[B32] Deicher R Ziai F Habicht A Bieglmayer C Schillinger M Horl WH Vitamin C plasma level and response to erythropoietin in patients on maintenance haemodialysis Nephrol Dial Transplant 2004 19 9 2319 2324 10.1093/ndt/gfh260 15299098

[B33] Keven K Kutlay S Nergizoglu G Erturk S Randomized, crossover study of the effect of vitamin C on EPO response in hemodialysis patients Am J Kidney Dis 2003 41 6 1233 1239 10.1016/S0272-6386(03)00356-1 12776276

[B34] Smith CH Bidlack WR Interrelationship of dietary ascorbic acid and iron on the tissue distribution of ascorbic acid, iron and copper in female guinea pigs J Nutr 1980 110 7 1398 1408 7381603 10.1093/jn/110.7.1398

[B35] Tomson CR Channon SM Parkinson IS McArdle P Qureshi M Ward MK Laker MF Correction of subclinical ascorbate deficiency in patients receiving dialysis: effects on plasma oxalate, serum cholesterol, and capillary fragility Clin Chim Acta 1989 180 3 255 264 10.1016/0009-8981(89)90007-7 2743578

[B36] Pru C Eaton J Kjellstrand C Vitamin C intoxication and hyperoxalemia in chronic hemodialysis patients Nephron 1985 39 2 112 116 10.1159/000183353 3974772

[B37] Bienfait HF van den Briel ML Rapid mobilization of ferritin iron by ascorbate in the presence of oxygen Biochim Biophys Acta 1980 631 3 507 510 10.1016/0304-4165(80)90028-8 7407258

